# Diversity of Cationic Antimicrobial Peptides in Black Cumin (*Nigella sativa* L.) Seeds

**DOI:** 10.3390/ijms24098066

**Published:** 2023-04-29

**Authors:** Anna S. Barashkova, Alexey N. Smirnov, Elena S. Zorina, Eugene A. Rogozhin

**Affiliations:** 1 Laboratory of Neuroreceptors and Neuroregulators, Shemyakin and Ovchinnikov Institute of Bioorganic Chemistry, RAS, 117437 Moscow, Russia; barashkova.an@gmail.com; 2 Laboratory of Biochemistry and Ecology of Microorganisms, All-Russian Institute for Plant Protection, 196608 Pushkin, Russia; 3 Department of Plant Protection, Timiryazev Russian State Agrarian University, 127434 Moscow, Russia; smirnov@timacad.ru; 4 Orekhovich Institute of Biomedical Chemistry, 119121 Moscow, Russia; el.petrenko@bk.ru; 5 Papanin Institute for Biology of Inland Waters Russian Academy of Sciences, 152742 Borok, Russia

**Keywords:** antimicrobial peptides, defensins, lipid-transfer proteins, *Nigella sativa*, proteomic analysis, fractionation, primary structure, 3D modeling, antimicrobial activity

## Abstract

Black cumin (*Nigella sativa* L.) is known to possess a wide variety of antimicrobial peptides belonging to different structural families. Three novel antimicrobial peptides have been isolated from black cumin seeds. Two of them were attributed as members of the non-specific lipid transfer proteins family, and one as a defensin. We have made an attempt of using the proteomic approach for novel antimicrobial peptides search in *N. sativa* seeds as well. The use of a well-established approach that includes extraction and fractionation stages remains relevant even in the case of novel peptides search because of the lacking *N. sativa* genome data. Novel peptides demonstrate a spectrum of antimicrobial activity against plant pathogenic organisms that may cause economically important crop diseases. These results obtained allow considering these molecules as candidates to be applied in “next-generation” biopesticides development for agricultural use.

## 1. Introduction

Plants are facing a variety of environmental factors during their ontogenesis. Antimicrobial peptides (AMPs) represent an ancient molecular instrument that provides defense from biotic and abiotic stresses [1,2]. AMPs are known to participate in plant innate immunity. They participate in the molecular defense against phytopatogenic microorganisms and pests. Additionally, they are involved in physiological processes such as fertilization and fruit ripening [1,3,4]. Several peptides are shown to be a part of the defense against abiotic stress factors (cold, drought, and heavy metals) [1,3,5].

Plant AMPs represent a group of cysteine-rich, mainly cationic molecules. Cysteine residues form disulfide bonds that provide a compact structure. They are classified into eight families according to their cysteine motifs: thionins, defensins, lipid-transfer proteins, hevein-like peptides, α-hairpinins (hairpin-like peptides), snakins, knottins, and cyclotides. Actually, there is a range of unclassified peptides: proline-rich, glycine-rich, shepheridins, etc. [6,7,8]. AMPs have a wide spectrum of biological activity: direct antimicrobial action via disruption of bacterial or fungal cells, enzyme inhibition, ion channel blocking, and ribosome inactivating. Some AMPs are important food allergens. Plant AMP is considered an antibiotic, antitumor agent, and immunomodulator [9,10]. AMPs occur in every plant, but peptide composition varies depending on a plant species or a plant organ. The highest amount of AMPs was isolated from seeds [7]. Some plants and plant families are well-studied sources of peptides of a particular family. *Violaceae* and *Rubiaceae* are known to produce a widespectrum of cyclotides [11,12]. Representatives of the same peptide family are found in other families (*Cucurbitaceae*, *Fabaceae*, and *Poaceae*), but their diversity is significantly less [13]. *Santalaceae* (*Viscum* sp., *Phoradendron* sp., *Pyrularia pubera*, etc.) are well-known sources of thionins [14,15,16], but there is no information about other AMPs in representatives of this plant family. At the same time, such species as radish and other *Brassicaceae*, as well as most cereals (wheat, rice, corn, etc.), are known to possess an orchestra of AMPs most commonly from defensins, thionins, nsLTPs, and hevein-like peptides families in case of *Triticum kicharae* [17,18,19,20]. In this context, the further study of AMP diversity in one single plant remains relevant and might uncover the mechanisms of plant defense and signaling. Previously AMPs from defensins [21], thionins [22,23], and lipid-transfer proteins [24] families have been found in black cumin (*Nigella sativa* L.) seeds using a well-established technique of acidic extraction.

## 2. Results

### 2.1. Proteomic Analysis

**Figure 1:**
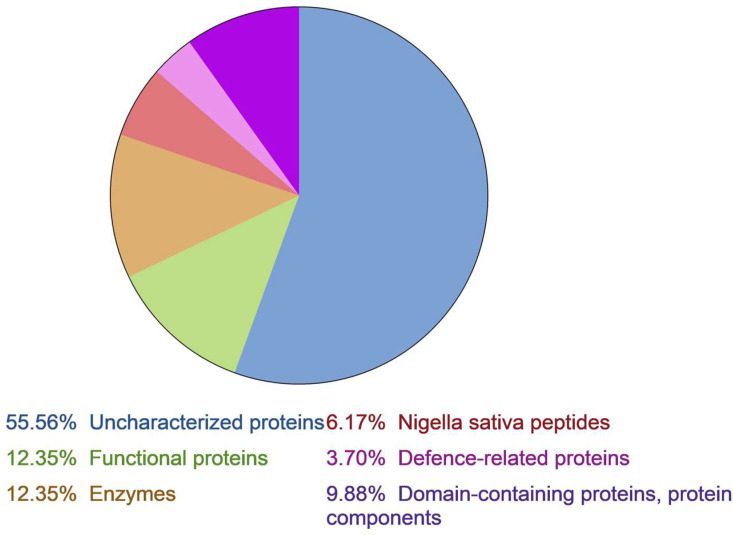
*Nigella sativa* proteome annotation.

First of all, a protein–peptide extract (PPE) from *N. sativa* flour has been obtained using the method that was previously applied [21,25]. The extraction scheme includes acidic extraction followed by precipitation with ice-cold acetone. As a result, proteins and peptides with positive surface charge are extracted. After that, PPE is desalted from polar low-molecular-weight metabolites via solid-phase extraction on a reversed-phase Aquapore HPLC C_8_ column (Applied Biosystems, Foster City, CA, USA) and concentrated. PPE was lyophilized and subjected to high-resolution mass spectroscopy (HRMS) to reveal proteomic content that is being found in interactive databases. This approach is valid and effective for high-throughput screening of different biological samples to evaluate a diversity of already known functional proteins, including antimicrobials. As a result, only previously isolated antimicrobial polypeptides were found amongst *Nigella sativa* (NCBI taxid: 555479), as well as other representatives of the genus *Nigella* (NCBI taxid: 3443). Their share did not exceed 11% ofall functional polypeptides identified and, consequently, less than 6% if uncharacterized proteins are included (Figure 1). In addition, other functional polypeptides are found to be revealed using more plants from the *Ranunculaceae* family, which have been deposited to GenBank/UniProt/SwissProt databases. Some proteins with high levelsof homology from *Aquilegia coerulea*, *Thalictrum thalictroides*, *Anemoclema glaucifolium*, and *Coptis chinensis* were found in the black cumin PPE. 

It was shown that 55.5% of proteins were identified as uncharacterized proteins, and about 38% of proteins are homologous to other plants’ enzymes, protein components and domains, and functional proteins (Figure 1). Some of them are related to plant immunity (thaumatin-like proteins, low temperature-induced proteins, programmed cell death proteins, etc.). Only 6% represent characterized antimicrobial peptides from black cumin. Therefore, this approach has demonstrated partial effectiveness as a tool to predict new AMPs (Supplementary File S1).

### 2.2. Peptide Isolation and Activity

**Figure 2:**
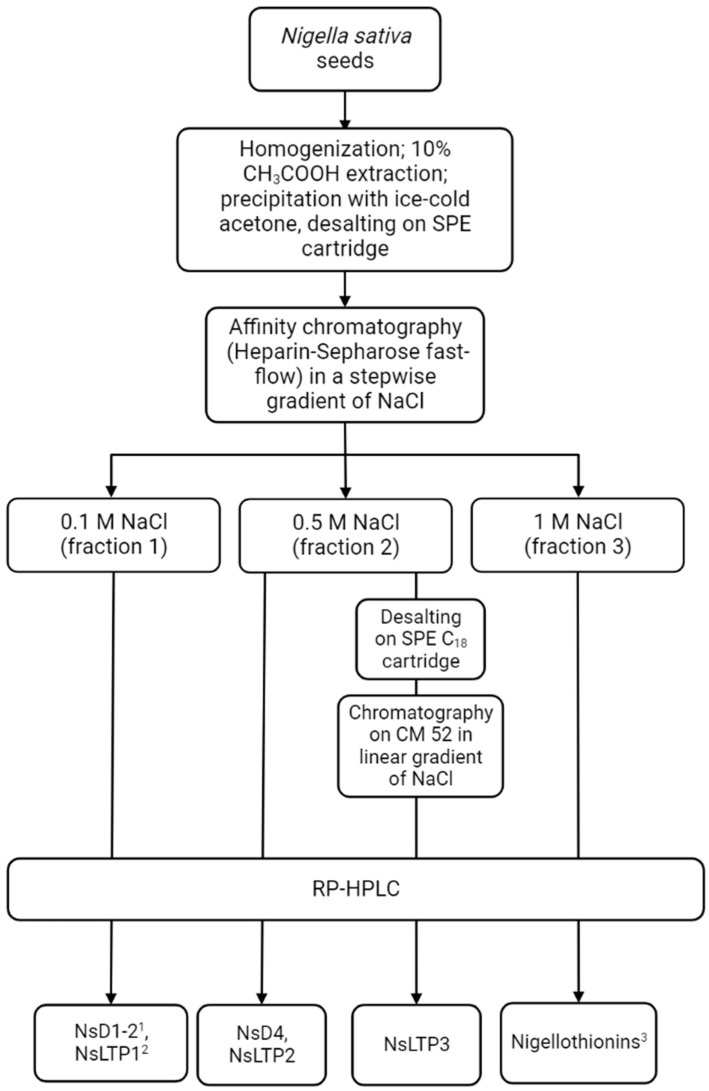
A flow diagram illustrated isolation and fractionation of black seed AMPs by liquid chromatography.^1^ [21]; ^2^ [24]; ^3^ [22,23].

Further fractionation was provided by a combination of liquid chromatography methods. In the first step, medium-pressure affinity chromatography on a Heparin-HiTrap Sepharose as a sorbent was carried out. Three fractions were obtained by a step-wise increase of NaCl concentration in the mobile phase. The fractions were collected at 0.1, 0.5, and 1 M of NaCl (Fraction 1, Fraction 2, and Fraction 3, respectively). The increase in NaCl concentration helps to fractionate PPE according to its ability to bind with heparin [26]. Fractions 1 and 3 were carefully studied during previous research. A number of AMPs from LTPs, defensins, and thionins families were isolated and characterized [21,23,24]. Here, we focused on the 0.5 M NaCl (Fraction 2) that was still uncovered. A flow diagram illustrating a modified fractionating approach is presented in Figure 2.

#### 2.2.1. Novel Defensin NsD4: Isolation and Structure Identification

**Figure 3:**
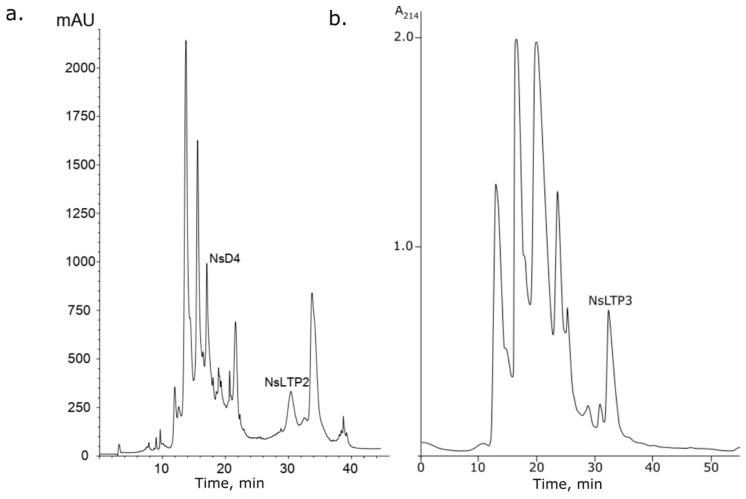
Isolation of individual peptides from the “fraction 2” after affinity chromatography. (**a**) RP-HPLC profile of fraction 2. (**b**) Cation-exchange chromatography profile of fraction 2.

Fraction 2 (0.5 M NaCl fraction) was subjected to analytical reversed-phase HPLC (RP-HPLC). The components of the mixture were collected manually. A component with a retention time of 17.1 min was characterized by MALDI-TOF MS and automated Edman sequencing (Figure 3a).

**Figure 4:**
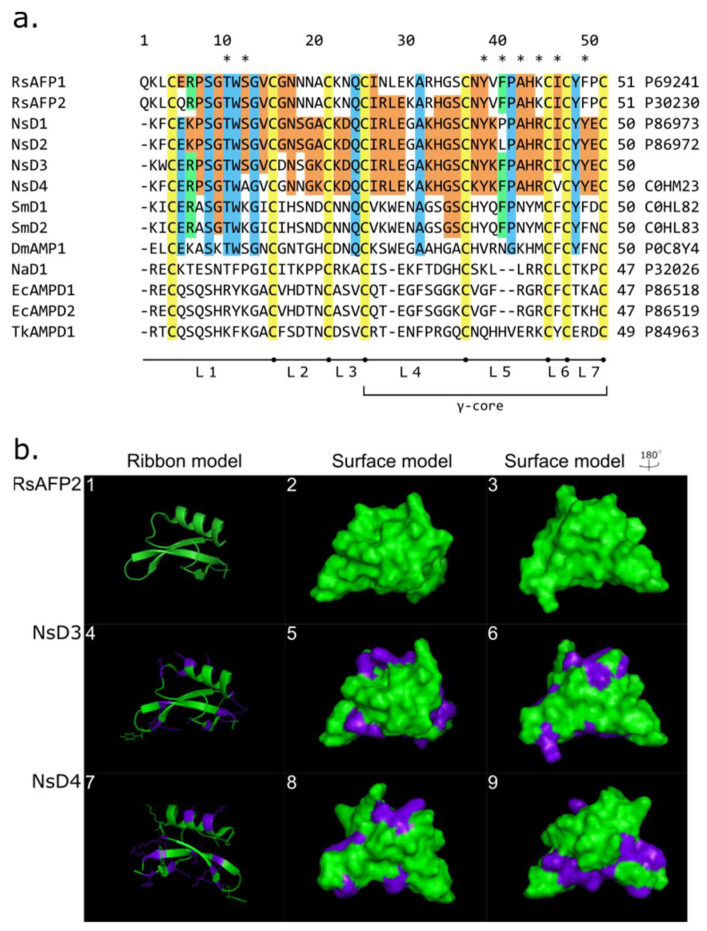
(**a**) Multiple alignments of *N. sativa* defensins sequences. Cysteine residues are marked in yellow; homologous residues in NsD1–4 are marked in orange; amino acid residues represented in other molecules as well as in NsDs are marked in blue; conserved substitutions are marked in green; amino acid residues responsible for antifungal activity are marked by asterisks; L1–7 indicated loops; γ-core region is indicated by a bracket; (**b**)modeling of three-dimensional structures of NsD3 (numbers 4–6) and NsD4 (numbers 7–9) in comparison to the RsAFP2 defensin structure (numbers 1–3).

It has been found that the novel peptide has an average molecular mass of 5697.1 Da. The complete 50-amino acid sequence (^1^KFCERPSGTWAGVCGNNGKCKDQCIRLEKAKHGSCKYKFPAHRCVCYYEC^50^) was determined via “non-stop” analysis. It was found that this peptide carries eight cysteine amino acid residues that form four disulfide bridges. According to a high level of homology to *N. sativa* defensins (NsD1/2) [21], it was attributed as a novel member of the defensins family and called NsD4 (UniProt ID: C0HM23). Previously, another defensin has been deduced using a full-length cDNA synthesized on total mRNA isolated from *N. sativa*. This molecule was called NsD3 (the corresponding cDNA was deposited to GenBank (ID: KX013490.1)). We can assume that *N. sativa* defensins represent a closely homologous group of peptides. The remarkable trait of NsD4 is the higher calculated level of surface charge under neutral pH compared with NsD1–3 group. It might be related to the presence of ε-amino groups of side chains of a lysine residue (Gly30Lys and Asn37Lys). It is worth noting that NsD3 and NsD4 have a hydrophobic residue (Phe) in position 39, as well as NsD2 (Figure 4a). It allows proposing a higher level of antifungal activity in contrast to NsD1. It was confirmed by modeling spatial structures. *Raphanus sativus* defensin RsAFP2, which is the most similar to NsDs, has been considered as a reference structure (Figure 4b). The gene encoding NsD3 was applied to construct transformed potato lines. Potato lines that carried the *nsd3* gene demonstrated higher resistance levels to a complex of fungal and bacterial diseases in laboratory and field experiments [27]. This can appear as indirect evidence of NsD3 antimicrobial activity, which is consistent with defensin’s nature. Three-dimensional modeling demonstrates the spatial structure change in NsD3–4 in comparison with RsAFP2 (Figure 4b). Amino acid substitutions are exposed to outer space. This allows considering their contribution to the peptide activity.

#### 2.2.2. Isolation and Identification of Novel Lipid-Transfer Proteins NsLTP2 and NsLTP3

**Figure 5:**
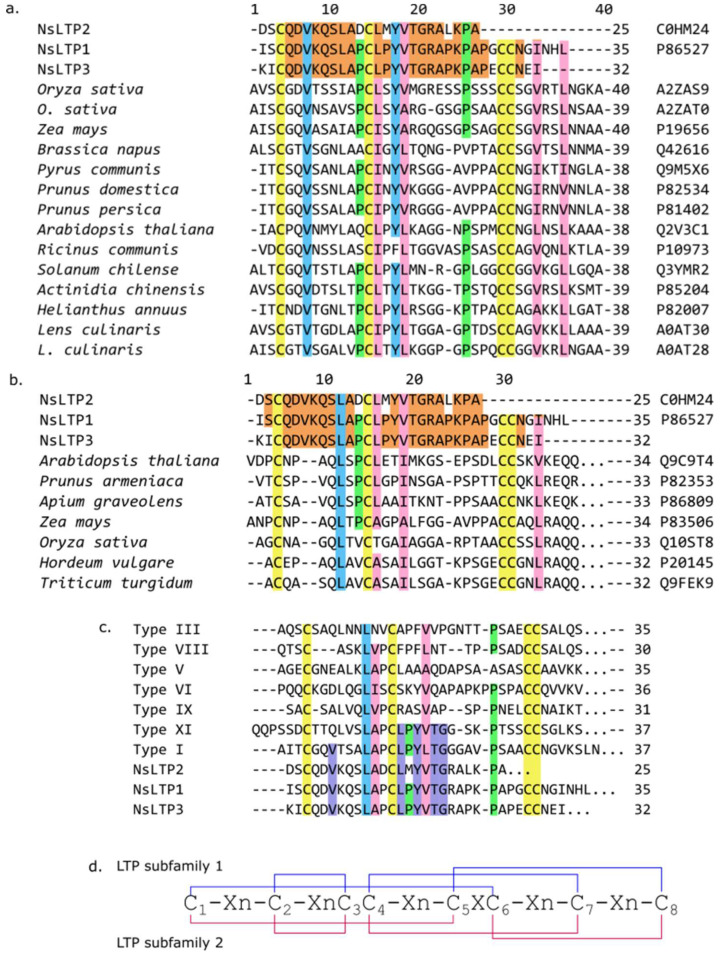
Multiple alignments of nsLTPs from *N.sativa* and other plants: (**a**) alignment of nsLTPs from *N.sativa* with various LTPs from structural subfamily 1; (**b**) alignment of nsLTPs from *N.sativa* with various LTPs from structural subfamily 2; (**c**) alignment of NsLTPs with consensus sequence alignment for all nsLTP types according to Fleury et al. [29]; (**d**) Cys-motifs and disulfide bonding of nsLTPs from structural subfamilies 1 and 2 [3]. Cys-residues are marked in yellow, Pro-residues are marked in green, homologous residues in NsLTP1–3 are marked in orange, amino acid residues represented in all nsLTPs considered are marked in blue, conservative amino acid substitutions are marked in pink, and residues that are similar inNsLTPs and nsLTPs from Types I and IX are marked in purple.

Another two peptides were isolated from fraction 3. The first one was isolated during RP-HPLC simultaneously with defensin NsD4, and its retention time was 30.4 min (Figure 3a). The second peptide was isolated during medium-pressure cation-exchange liquid chromatography (MPLC) on CM52 of fraction 3 (Figure 3b). Both compounds were also characterized by MALDI-TOF MS and N-terminal sequencing. Their average molecular masses were 9392.4 and 5707.3 Da, respectively. Partial N-terminal amino acid sequences (up to 25 residues) were estimated by automated Edman degradation: ^1^DSCQDVKQSLADCLMYVTGRALKPA^25^… for the first peptide and ^1^KICQDVKQSLAPCLPYVTGRAPKPA^25^… for the second one. Based on sequence homology in BLASTP searching, these molecules were attributed to the 9-kDa non-specific lipid transfer protein subfamily [28] (Figure 5a). These peptides were called NsLTP2 (UniProt ID: C0HM24) and NsLTP3 (UniProt SPIN ID 200024576), and they have a high homology in their N-terminal sites and make local differences through variable amino acid substitutions (Asp1Lys, Ser2Ile, Asp12Pro, Met15Pro, and Leu22Pro) (Figure 5a). It is interesting to point out that the N-terminal fragment for NsLTP3 is Pro-enriched. Most proline residues are associated with the first and the second α-helices, which could make them bent [3]. Moreover, this molecule is attractive for further study since it has a lower measured molecular mass (about 6 kDa instead of 9 kDa for NsLTP2 and NsLTP1 [24]), whereas these two polypeptides are very homologous.

### 2.3. NsLTP3 Antimicrobial Activity

#### 2.3.1. Antifungal Activity

With regard to unusual structural characteristics, the NsLTP3 peptide was selected for deep evaluation of the antimicrobial activity. First, this AMP was tested against filamentous phytopathogenic fungi using a micro-dilution assay. Four peptide concentrations were tested (2.1–0.26 µM). Peptide activity level was measured as IC_50_ (peptide concentration that inhibited conidial germination up to 50%).It was measured according to thepercent of germinated conidia. The integrity of cell structures in response to peptide action was estimated as well. NsLTP3 was active against all filamentous fungi tested. *A. niger* VKM F-33 has demonstrated the highest level of sensitivity: its IC_50_ level was 1.05 µM. *B. cinerea* TSKHA isolated from damaged potato was the less sensitive (IC_50_ = 1.88 µM). *B. sorokiniana* VKM F-1448 has demonstrated moderate sensitivity (IC_50_ = 1.55 µM), but plasmolysis has been detected.

#### 2.3.2. Antioomycetal Activity

Antioomycetal activity of NsLTP3 was monitored ex vivo on potato tuber discs. Zoosporangia were incubated with peptide (0.525–4.2 µM) and applied on potato disc surfaces. Peptide activity was estimated according to the infected area from “++++”—total inhibition of infectious process to “–”—the absence of inhibition. Two *P. infestans* strains were taken to the experiment. *P. infestans* OSV12 represents an aggressive variety, and *P. infestans* PRIL is the less aggressive one. It was shown that none of the peptide concentrations taken did not provide total inhibition of disease development. The more aggressive strain *P. infestans* OSV12 appeared to be more sensitive to the peptide action. At the same time, the less aggressive *P. infestans* PRIL seemed to retain the ability to cause disease up to 144 h at the lowest peptide concentration (Table 1, Supplemental Figure S1).

#### 2.3.3. Antibacterial Activity

NsLTP3 was tested against phytopathogenic bacteria via radial diffusion assay. Peptide was active against all bacteria tested (Gram-positive as well as against Gram-negative). The highest activity level has been detected against Gram-negative *X. campestris* and Gram-positive *B. subtilis*; the IC_50_ levels were 7.0 ± 0.5 µM and 8.4 ± 1.0 µM, respectively. It also has demonstrated activity against Gram-negative *P. carotovorum* and and *P. syringae* with IC_50_ levels of 11.5 ± 1.3 and 12.0 ± 1.4 µM. The activity against Gram-positive *C. michiganensis* sb sp. *michiganense* was comparable (IC_50_ level 11.2 ± 1.1 µM). Another lipid-transfer protein from *N.sativa* seeds—NsLTP1 was tested as well. It has been shown that NsLTP1 has a narrow spectrum of antibacterial activity. It was inactive against *X. campestris*, *P. carotovorum*, and *C. michiganensis* sb. sp. *michiganense*. In the case of *Ps.syringae* and *B. subtilis*, its activity level was comparable to NsLTP3 (IC_50_ levels 10.0 ± 1.0 µM and 7.0 ± 0.5 µM, respectively) (Supplementary File S2).

## 3. Discussion

It has been shown that *N. sativa* has become a source of a wide variety of antimicrobial peptides from different structural families: defensins, thionins, and LTPs [21,23,24]. All of them were isolated using the same extraction technique that covers a couple of extraction procedures and a series of liquid chromatography stages [25]. The occurrence of such a variety of AMPs from different structural families allows assuming that *N. sativa* can probably store a wider diversity of AMPs, including novel molecules. It drives to further search forpeptides in this plant. At present, the genome-wide approach is used to discover predicted AMPs in well-studied plants such as mouse-ear cress (*Arabidopsis thaliana*), rice (*Oryza sativa*), cotton (*Gossypum hirsutum*), wheat (*Triticum* spp.), barrel clover (*Medicago truncatula*), etc. [19,30,31,32,33]. This approach might be good for the evaluation of the full spectrum of NsAMPs, but its genome is still unraveled, and its approximate size is over 6 Gb and now is still being estimated [BioProject NCBI ID: PRJNA686272]. So, we confidently consider that the “wet” approach remains relevant for such plants lacking genome data. In this study, we attempted to apply the proteomic approach for AMPs search, prediction, and identification. Unfortunately, this approach did not allow us to find or to predict any novel peptides even after the expansion of the search of *Ranunculaceae* family level.

Thus, we have applied a well-established scheme for novel AMPs search. Based on a combination of liquid chromatography coupled with mass-spectrometry and N-terminal sequencing a novel representative of *N. sativa* defensins was isolated. NsD4 demonstrates high homology to radish (*R. sativus*) defensins RsAFP1–2. It demonstrates a high similarity to RsAFP2 in the gamma-core region (loops 4–7) especially in loop 5,where 7 of 10 essential for antifungal activity amino acid residues are placed (Figure 4b). Moreover, NsD4 carries Thr10, Tyr38, Phe40, and Ala42 residues that are responsible for antifungal activity in RsAFPs [34]. It also contains conservative Lys44Arg and Phe49Tyr substitutions. Another essential amino acid residue, serine in the 12th position, is substituted with alanine (Figure 4b).

*N. sativa* defensins demonstrate a high level of sequence similarity with antifungal defensins from group 9 according to van der Weerden and Andersen [35], which includes antifungal defensins, some of which can initiate hyper branching in fungi. The most well-characterized representative of this group is radish defensin RsAFP, which is considered a typical representative. Predicted peptide NsD3 carries six of eight amino acid residues essential for antifungal activity presented in NsD3 (Thr10, Ser12, Tyr38, Phe40, Ala42, and Ile46); also, there are two conserved substitutions, Lys44Arg and Phe49Tyr, which allows us to considerthe high level of antifungal activity (Figure 4b). This consideration has been supported by data obtained on potato transgenic lines expressing the *nsd3* gene. Transgenic potato has shown resistance level to a complex of bacterial and fungal pathogens [27]. Four of eight essential amino acid residues are presented in NsD4 (Thr10, Tyr38, Phe40, and Ala42).

It is worth noting that defensins NsD1/2 havea similar substitution pattern as NsD3. It has been shown that Ser12Arg substitution in RsAFP2 leads to a significant decrease in antifungal activity against *Fusarium culmorum*. This allows us to consider that NsD4 might be less active than NsD1–3 or RsAFP2 (IC_50_ on *F. culmorum* 0.4 µM) [18].

Defensins from *N. sativa* possess a slight level of homology with SmD1–2 from common chickweed (*S. media*). The level of similarity varies from 48% for NsD1–2 to 52 and 50% for NsD3 and NsD4, respectively. This is consistent with their activity levels on different *Fusarium* species. IC_50_ of NsD1/D2 was of 0.5–1.7/0.32–1.7 µM and SmD1/2 had IC_50_ of 0.35–0.5 µM on the same species [21,36]. At the same time, *N. sativa* defensins demonstrate low sequence similarity levels with other antifungal peptides such as hairpin-like EcAMPs from barnyard grass (*E. crus-galli*), NaD1 from ornamental tobacco (*Nicotiana alata*), and Tk-AMP-D1 from wheat (*T. kiharae*) (Figure 4b). This illustrates their relation to the RsAFPs group.

Novel representatives of the *N. sativa* non-specific lipid-transfer proteins family have also been isolated using the above-mentioned method. Two novel peptides were isolated and characterized partially. According to the phylogenic nsLTPs classification, proposed by Fleury et al. [29], novel NsLTP2 and NsLTP3 peptides share the highest degree of similarity to nsLTPs from subfamilies I and XI (Figure 5c). According to the classification mentioned type I, nsLTPs represent a huge group that includes all peptides from structural subfamily 1 (9-kDa, carrying a “hydrophobic tunnel”, Figure 5d). The high level of homology of NsLTPs allows us to consider the novel peptides as members of structural subfamily 1 (type I) of nsLTPs (Figure 5a). Sequence similarity of novel peptides to nsLTPs from structural subfamily 2 (7-kDa, carrying a “hydrophobic cleft”, Figure 5d) is low (Figure 5b).

The novel NsLTP2 and NsLTP3 from *N. sativa* possess a high level of antimicrobial activity against pathogenic microorganisms. Antifungal activity of NsLTP3 against *B. cinerea* and *A. niger* was about 5timeshigher than for LTPs from radish (*R. sativa*) and from mother worth (*Leonorus japonicus*) and up to 18 times higher than nLenc3 from lentil (*L. culinaris*) [37,38,39]. It has been found that NsLTP3 causes plasmolysis in *B. sorokiniana*, which corresponds to one of the proposed mechanisms of nsLTPs antimicrobial activity related to membrane-permeabilizing properties [40].

Novel peptides demonstrate a high level of antibacterial activity against Gram-positive and Gram-negative bacteria which is of special importance because of the lack of anti-Gram^−^ agents. However, the level of activity of NsLTPs was relatively low. It has been shown that MsLTP_1_ from noni seeds inhibited the growth of *Staphylococcus aureus* and *S. epidermidis* at a concentration of 12.5 µg/mL (about 1.4 µM), but it was inactive against Gram-negative human pathogens [41]. At the same time, nsLTPs from barley (*H. vulgare*) have shown a remarkable activity level against plant Gram-negative pathogenic bacteria *P. solanacerum* and *C. michiganensis* subsp. *sepedonicus*—3–6 and 1–3 µM, respectively [42]. It should be noted that a relatively low level of NsLTP2–3 antibacterial activity remains of interest because the ability to inhibit the growth of Gram-negative bacteria is rare. Some of nsLTPs, e.g., Ps-LTP1 from pea (*Pisum sativum*), do not inhibit the growth of Gram-negative bacteria [43].

Non-specific lipid transfer proteins are found to represent a family of plant pathogenesis-related proteins class 14 (PR-14). They participate in plant defense from biotic and abiotic stress. It is known that overexpression of LTPs genes enchases plant resistance to pathogens attack [44,45]. Additionally, LTP gene knock-out leads to systemic acquired resistance mechanism disruption [46]. It has been shown that LTPs play a significant role in plant signaling during plant–pathogen interaction. Overexpression of potato LTP StLTP10 increases the resistance of transgenic potatoes toward *P. infestans* in vivo [47]. Nevertheless, it is still less known about the direct interaction of LTPs with oomycetes. Plant peptide extracts and preparations may inhibit *P. infestans* growth and germination [48,49]. By inducing plant defense systems or direct interaction with pathogens. NsLTPs demonstrate a higher level of inhibition on *P. infestans* ex vivo at the first 96 h of incubation than LTPs from barnyard grass (*E. crus-galli*) seeds [50]. The study of the antioomycetal activity of cheese weed (*Malva parviflora*) has shown the inhibition of *P. infestans* growth in in vitro experiments [51]. That evidence provides the consideration that participation in plant signaling is not the only way of antifungal action of nsLTPs.

## 4. Materials and Methods

### 4.1. Plant Material

Seeds of *Nigella sativa* L. (variety “Krymchanka”) were collected in the Republic of Crimea in 2019.

### 4.2. Peptide Extraction

Peptides wereextracted from *N. sativa* seeds using the procedure which was applied for plant antimicrobial peptideextraction before [21,22,25]. Briefly, seeds were powdered in coffee meal and extracted with ten volumes of 10% acetic acid supplied with 10 µL/L of antiprotease cocktail (Sigma-Aldrich, Burlington, MA, USA) for one hour on magnetic stirrer. Seed debris was separated using gauze, and the resulting extract was centrifuged (6000 rpm, 10 min, 4 °C). Supernatant was collected into a cylinder, and ice-cold acetone was added at 1 to 7 ratio to precipitate proteins and peptides extracted and stayed overnight at 4 °C. After protein–peptide extract (PPE) precipitation liquid was removed manually. Air-dried precipitate was suspended in 0.1% trifluoracetic acid (TFA), desalted using solid phase extraction C_18_ cartridge, and lyophilized (Figure 2).

### 4.3. Affinity Chromatography

Affinity chromatography was carried out on Heparin-HiTrap Sepharose 5 mL (GE HealthCare, Chicago, IL, USA) using 10 mM Tris-HCl pH 7.2 (Solvent A) and 10 mM Tris-HCl supplied with 1 M NaCl pH 7.2 (Solvent B) as a mobile phase. Lyophilized PPE was dissolved in 10 mM Tris-HCl buffer pH 7.2 and applied on the column pre-equilibrated with Solvent A. Stepwise elution with 0.1 (fraction 1), 0.5 (fraction 2), and 1.0 M (fraction 3) NaCl was used to fractionate PPE components according to their surface charge (Figure 1). Fractions were collected manually and desalted.

### 4.4. Cation Exchange Chromatography

Fraction 2 after affinity chromatography was desalted and subjected to cation exchange chromatography on carboxymethyl cellulose 52 (CM52). Fraction 2 was lyophilized and redissolved in 50 mM Tris-HCl (pH 7.2) and applied toa column filled with carboxymethyl cellulose 52 (CM52). Fractionation was carried out in a linear gradient of NaCl (0–90% within 90 min) in 50 mM Tris-HCl. 

### 4.5. Reversed-Phase HPLC 

Final purification of the peptides was performed by RP-HPLC on a ReproSil-Pur 300 ODS-3(4.6 × 250 mm, 5 microns, 300 Å “Dr. A. Marsch Ammerbuch”, Tübingen, Germany) in a linear acetonitrile (Panreac Quimica, Barselona, Spain) gradient (from 10 to 50% B) (solvent B is 80% MeCn in addition of 0.1% TFA) for 1 h at a flow rate of 0.8 mL/min and 42 °C. Elution of peptides was monitored at 214 nm.

### 4.6. MALDI-TOF/TOF MS

Molecular masses of proteins and peptides were measured on an Ultraflex MALDI mass spectrometer (Bruker Daltonics, Billerica, MA, USA) in a positive ion mode. Mass spectra were recorded in an average mode (1–20 kDa). 2,5 dihydroxybenzoic acid was used as a matrix. Mass spectra were analyzed with Bruker DataAnalysis for TOF software (v. 4.0). The accuracy of mass determination was 0.015%.

### 4.7. 3D Structure Modeling

Modeling of defensin spatial structure was accomplished using PyMol v. 0.9.3 software.

### 4.8. Edman Sequencing

N-terminal amino acid sequencing was performed on an automated sequencer (PPSQ-33A model, Shimadzu Corporation, Kyoto, Japan) according to the manufacturer’sprotocol. Approximately 700–800 pmoles of each peptide was taken for analysis. Identification of amino acid residues (PTH-derivatives) was performed using LabSolutions software (v. 1.0.1) (Shimadzu Corporation, Kyoto, Japan).

### 4.9. Reduction and Alkylation

For reduction and alkylation of disulfide binds, 1 M dithioerythrol (DTET) and 50% (*v*/*v*) 4-vinylpyridine in 2-propanol were used. The procedure was carried out according to method described earlier [21].

### 4.10. Chemicals

All the chemicals used in the preparation of buffers and solutions were of analytical reagent grade or better. Sodium hydroxide(Sigma-Aldrich, Burlington, MA, USA) (NaOH, ≥99.0%, pellets), hydrochloric acid (HCl, 37% (*v*/*v*)), boric acid (H_3_BO_3_, ≥99.5%), acetonitrile (can, LC-MS grade), water (LC-MS grade), bovine serum albumin (BSA, relative molecular mass (Mr) of approximately 66,000), acetic acid (HAc, glacial), formic acid (FA, 99.0%), acetone (≥99.5%), ethanol (96.0%), glycerol (≥99.5%), tris (hydroxymethyl)aminomethane hydrochloride (Tris-HCl, ≥99.9%), sodium dodecyl sulfate (SDS, ≥99.8%), 4-(2-hydroxyethyl)-1-piper-azineethanesulfonic acid (HEPES, ≥99.5%), urea (≥99.0%), Triton™ X-100 (laboratory grade), tris(2-carboxyethyl)phosphine hydrochloride (TCEP, ≥98.0%), and iodoacetamide (IAA, ≥99.0%) were supplied by Merck (Darmstadt, Germany). β-mercaptoethanol (≥99.0%) was provided by PanReac Applichem (Barcelona, Spain). Trypsin/Lys-C enzyme mix (MS grade) was purchased from Promega (Madison, WI, USA). Bromophenol blue, tetramethylethylenediamine (TEMED, ≥99.0%), acrylamide/bis solution (30% (*v*/*v*)), ammonium persulfate (APS, ≥98.0%), and Bio-Safe™ Coomassie stain were supplied by Bio-Rad (Hercules, CA, USA). BenchMark™ Protein Ladder was provided by Thermo Fisher Scientific (Waltham, MA, USA). Water with conductivity lower than 0.05 μS/cm was obtained using a Milli-Q water purification system (Millipore, Molsheim, France).

### 4.11. Proteome Analysis

Peptidome analysis was carried according to [52]. Briefly, *N. sativa* PPE volume corresponding to 50 µg of total protein estimated by CE-UV was evaporated to dryness and suspended in 100 μL of ice-cold extraction buffer (25 mM HEPES, pH 8.0, 1.5 M urea, 0.02% (*v*/*v*) Triton™ X-100 and 5% (*v*/*v*) glycerol). Samples were reduced by addition of 3 mM TCEP for 45 min at RT and then alkylated with 15 mM IAA for 60 min in the dark at RT.

Proteolytic digestion was performed using trypsin/Lys-C mix (enzyme/protein ratio 1:167 m/m). Mixture was incubated overnight under shaking. The digestion was stopped withaddition of FA (1% (*v*/*v*) final concentration) and centrifuged. The supernatant containing the digested proteins was desalted and evaporated to dryness.

All experiments were performed on an Orbitrap Fusion™ Lumos™ (Thermo Scientific, Waltham, MA, USA) coupled to an Ultimate3000 nanoRLSC (Thermo Scientific, Waltham, MA, USA). Protein digests were dissolved in 20 μL of water containing 1% of FA (*v*/*v*) and separated on a column in-house packed with C18 particles (Luna C_18_(2), 3 μm, 100 Å, Phenomenex, Torrance, CA, USA) using a water/ACN/0.1% (*v*/*v*) FA linear gradient of ACN at a flow rate of 0.30 μL/min. A total of 2 μL of sample was injected. The Orbitrap parameters in ESI+ were as follows: ion source temperature 250 °C, ion spray voltage 2.1 kV, top speed mode, and full-scan MS spectra (*m*/*z* 350–2000) acquired at a resolution of 60,000. Precursor ions were filtered according to monoisotopic precursor selection, charge state (+2 to +7), and dynamic exclusion (30 s with a ±10 ppm window). The automatic gain control settings were 5 × 10^5^ for full scan and 1 × 10^4^ for MS/MS scans. Fragmentation was performed with collision-induced dissociation (CID) in the linear ion trap. Precursors were isolated using a 2 *m*/*z* isolation window and fragmented with a normalized collision energy of 35%.

Data analysis was carried out using MaxQuant (Thermo Scientific, Waltham, MA, USA, v. 1.6.17.0) (Cox & Mann, 2008) with the search engine Andromeda [53] applied for protein and peptide identification for all MS raw files. Enzymatic digestion with trypsin was selected, together with a maximum of two missed cleavages, peptide charges from +2 to +7, a precursor mass tolerance of 10 ppm, and a fragment mass tolerance of 0.5 Da. Search parameters were set to allow for dynamic modifications of methionine oxidation, acetyl on N-terminus, and fixed cysteine carbamidomethylation.

The search database consisted of a non-redundant *N. sativa* protein sequence FASTA file containing the 81 entries from *Ranunculaceae* family found in RefSeq NCBI database (FASTA file is provided as Supplementary Material). The false discovery rate (FDR) was set to 0.01 for both peptide and protein identifications. Normalized label-free quantification (LFQ) values were obtained by applying the in-built MaxLFQ algorithm [53]. MaxQuant normalized LFQ intensities of identified proteins in all quinoa varieties were visualized as a heat map, created using the freely available web server Heatmapper (http://www.heatmapper.ca) accessed on 15 December 2021. The identified proteins were also classified by Gene Ontology (GO) using the PANTHER classification system (http://www.pantherdb.org (accessed on 15 December 2021)). However, as *Nigella sativa* is not available in the PANTHER-GO system, which works primarily with UniProt identifiers and modeled organisms, the NCBI accession numbers (IDs) of the identified proteins were blasted against the Uniprot database of *Ranunculaceae* family.

### 4.12. Antimicrobial Assay

#### 4.12.1. Microorganisms

*Aspergillus niger* VKM F-33 and *Bipolaris sorokiniana* VKM F-1448 were purchased in All-Russian Collection of Microorganisms G.K. Skryabin Institute Biochemistry and Physiology of Microorganisms Russian Academy of Sciences (Pushchino, Moscow region, Russia); *Botrytis cinerea* TSKHA was isolated from damaged potato plants and were kindly supplied by the Department of Plant Protection K.A Timiryazev Russian State Agrarian University (Moscow, Russia). *Phytophtorainfestans* strain OSV 12 (aggressive strain) and *Ph.infestans* PRIL (less aggressive strain) were obtained from the Institute of Plant Protection of the Republic of Belarus. Bacterial strains *Pseudomonas syringae*, *Xanthomonas campestris*, *Pectobacterium carotovorum*, *Bacillus subtilis*, and *Clavibacter michiganense* sb. sp. *michiganense* were supplied by the Collection of the Vavilov Institute of General Genetics of the Russian Academy of Sciences (Moscow, Russia).

#### 4.12.2. Antibacterial Assay

Petri dishes with Luriae-Bertani agar were seeded with bacterial suspensions. Solutions of peptides (50 mL) were applied into 5 mm diameter wells punched in agar media. After applying peptides, Petri dishes were incubated at room temperature for 48 h. The antibacterial activity was evaluated according to the diameters of inhibition zones formed around the wells with the peptide solution [21]. Final peptide concentration was 50 µg (8.8 nmol) per well. Kanamycin (10 μg/well) has been considered as a positive control and water—as a negative control.

#### 4.12.3. Antifungal Activity In Vitro

Antifungal activity of NsD4 was determined, as given in [54], with some modifications. Fungal colonies were incubated at 25 °C for 8–10 days. Conidia were washed from the surface of mycelium with 10 mL of potatodextrose broth (Sigma-Aldrich, Burlington, MA, USA) and diluted to a concentration of ~2 × 10^4^ CFU/mL.Two-fold peptide dilutions were placed in microtiter plates (BioCell, Miami, FL, USA) containing two-fold serial dilutions. Fungal suspensions were placed into the wells of the peptide (from 1.25 to 10.0 mM). Incubation with peptides proceeded at 25 °C for 48 h. Inhibition of spore germination was examined by light microscopy using an Axio Skope A1 instrument (Carl Zeiss, Oberkochen, Germany) at 180-fold magnification in 10 visual fields. The degree of inhibition was calculated as the percentage of germinated conidia against their total number. The IC_50_ values were calculated as the peptide concentration that caused 50% inhibition of spore germination. Inhibition of hyphal elongation and morphological changes in the fungi were also examined at 360-fold magnification. The experiment was carried out in three replicates.

#### 4.12.4. Antioomycetal Activity Ex Vivo

Biological activity of peptide extracts was estimated according to the degree of *Phytophthora infestans* development inhibition *ex vivo* using potato tuber discs [48]. Two similar-sized potato tuber discs were placed in Petri dishes. Peptide samples were mixed with 50 µL of zoosporangium suspension (5000 zoosporangia mL^−1^) to a final peptide extract concentration of 0.125–2.0 mg/mL and incubated at 20 °C for 2 h. Preincubated mixture of peptide with zoosporangiawas applied to the center of potato tuber disc. Potato discs infected with the zoosporangium suspension without peptide served as a control. The Petri dishes with infected potato tuber discs were incubated at 20 °C for 120 h. The development of infection was estimated after 96, 120, and 144 h of incubation according to the infection area. The severity of disease on potato tuber discs caused by *P. infestans* was estimated regarding control and scored from “−” to “++++”, with “++++” denoting complete inhibition of disease symptoms, “+++” disease development less than 10%, “++” disease development less than 20%, “+”disease development less than 40% “−” disease development 40% (the absence of inhibition). The infected area was measured using Abode Photoshop for Windows 7 Ultimate software. Ten discs were analyzed in each of three independent experiments.

## 5. Conclusions

Herein, three novel antimicrobial peptides from the plant defensin and the lipid transfer proteins families were characterized. They were isolated from black cumin (*N. sativa*) seeds based on liquid chromatography techniques. Structural analysis was conducted via analytical methods (MALDI MS and Edman sequencing). Proteomic analysis was carried out to reveal novel potential AMPs that could be found in *N. sativa* seeds extract, but it was unsuccessful, and results were unclear because there are no reference amino acid sequences enough among the *Nigella* genera, as well as *Ranunculaceae* family in whole. The isolated peptides possess antimicrobial properties that were estimated by similarity and experimentally. According to the data obtained, *N. sativa* is considered a wealthy reservoir of AMPs belonging to different structural families and displaying diverse modes of action. Fundamentally, this option provides this plant with a strong defense from biotic stress factors like fungal and bacterial diseases.

## Figures and Tables

**Table 1:** Inhibitory activity of NsLTP3 on disease development caused by *P. infestans* OSV12/PRIL (more aggressive/less aggressive). “+++” disease development less than 10%, “++” disease development less than 20%, “+”disease development less than 40% “−” the absence of inhibition.

NsLTP3 Concentration, µM	Time of Incubation, h		
	96	120	144
4.2	+++/+++	++/++	+/++
2.1	++/+++	++/++	+/+
1.05	++/+++	++/++	−/+
0.525	++/++	+/+	−/+

## Data Availability

The sequence data will be available in the UniProt database: NsD4 C0HM23; NsLTP2 C0HM24, NsLTP3 UniProt SPIN ID 200024576. cDNA of NsD3 is available in the GenBank database ID: KX013490.1. Proteomic data are available via ProteomeXchange with the identifier PXD040611.

## Supplementary Materials

The following supporting information can be downloaded at: https://www.mdpi.com/article/10.3390/ijms24098066/s1.

## References

[1] Parisi K., Shafee T.M.A., Quimbar P., van der Weerden N.L., Bleackley M.R., Anderson M.A. (2019). The evolution, function and mechanisms of action for plant defensins. Semin. Cell Dev. Biol..

[2] Höng K., Austerlitz T., Bohlmann T., Bohlmann H. (2021). The thionin family of antimicrobial peptides. PLoS ONE.

[3] Finkina E.I., Melnikova D.N., Bogdanov I.V., Ovchinnikova T.V. (2016). Lipid transfer proteins as components of the plant innate immune system: Structure, functions, and applications. Acta Nat..

[4] Finkina E.I., Melnikova D.N., Bogdanov I.V., Ovchinnikova T.V. (2018). Peptides of the Innate Immune System of Plants. Part I. Structure, Biological Activity, and Mechanisms of Action. Russ. J. Bioorg. Chem..

[5] Tang S.S., Prodhan Z.H., Biswas S.K., Le C.F., Sekaran S.D. (2018). Antimicrobial peptides from different plant sources: Isolation, characterisation, and purification. Phytochemistry.

[6] Li J., Hu S., Jian W., Xie C., Yang X. (2021). Plant antimicrobial peptides: Structures, functions, and applications. Bot. Stud..

[7] Finkina E.I., Melnikova D.N., Bogdanov I.V., Ovchinnikova T.V. (2019). Peptides of the Innate Immune System of Plants. Part II. Biosynthesis, Biological Functions, and Possible Practical Applications. Russ. J. Bioorg. Chem..

[8] Tam J.P., Wang S., Wong K.H., Tan W.L. (2015). Antimicrobial peptides from plants. Pharmaceuticals.

[9] Campos D.C.O., Costa A.S., Lima A.D.R., Silva F.D.A., Lobo M.D.P., Monteiro-Moreira A.C.O., Moreira R.A., Leal L.K.A.M., Miron D., Vasconcelos I.M. (2016). First isolation and antinociceptive activity of a lipid transfer protein from noni (*Morinda citrifolia*) seeds. Int. J. Biol. Macromol..

[10] Skypala I.J., Bartra J., Ebo D.G., Antje Faber M., Fernández-Rivas M., Gomez F., Luengo O., Till S.J., Asero R., Barber D. (2021). The diagnosis and management of allergic reactions in patients sensitized to non-specific lipid transfer proteins. Allergy.

[11] Ovesen R.G., Brandt K.K., Göransson U., Nielsen J., Hansen H.C.B., Cedergreen N. (2011). Biomedicine in the environment: Cyclotides constitute potent natural toxins in plants and soil bacteria. Environ. Toxicol. Chem..

[12] Burman R., Gruber C.W., Rizzardi K., Herrmann A., Craik D.J., Gupta M.P., Göransson U. (2010). Cyclotide proteins and precursors from the genus Gloeospermum: Filling a blank spot in the cyclotide map of Violaceae. Phytochemistry.

[13] Huang Y.H., Du Q., Craik D.J. (2019). Cyclotides: Disulfide-rich peptide toxins in plants. Toxicon.

[14] Vernon L.P., Evett G.E., Zeikus R.D., Gray W.R. (1985). A toxic thionin from *Pyrularia pubera*: Purification, properties, and amino acid sequence. Arch. Biochem. Biophys..

[15] Tsekouras V., Mavrikou S., Vlachakis D., Makridakis M., Stroggilos R., Zoidakis J., Termentzi A., Moschopoulou G., Kintzios S. (2020). Proteome analysis of leaf, stem and callus in Viscum album and identification of lectins and viscotoxins with bioactive properties. Plant Cell Tissue Organ Cult..

[16] Johansson S., Gullbo J., Lindholm P., Ek B., Thunberg E., Samuelsson G., Larsson R., Bohlin L., Claeson P. (2003). Small, novel proteins from the mistletoe Phoradendron tomentosum exhibit highly selective cytotoxicity to human breast cancer cells. Cell Mol. Life Sci..

[17] Odintsova T.I., Egorov T.A., Musolyamov A.K., Odintsova M.S., Pukhalsky V.A., Grishin E.V. (2007). Seed defensins from T. kiharae and related species: Genome localization of defensin-encoding genes. Biochimie.

[18] Terras F.R.G., Schoofs H.M.E., De Bolle M.F.C., Van Leuven F., Rees S.B., Vanderleyden J., Cammue B.P.A., Broekaert W.F. (1992). Analysis of two novel classes of plant antifungal proteins from radish (*Raphanus sativus* L.) seeds. J. Biol. Chem..

[19] Boutrot F., Chantret N., Gautier M.F. (2008). Genome-wide analysis of the rice and arabidopsis non-specific lipid transfer protein (nsLtp) gene families and identification of wheat nsLtp genes by EST data mining. BMC Genom..

[20] Odintsova T.I., Vassilevski A.A., Slavokhotova A.A., Musolyamov A.K., Finkina E.I., Khadeeva N.V., Rogozhin E.A., Korostyleva T.V., Pukhalsky V.A., Grishin E.V. (2009). A novel antifungal hevein-type peptide from Triticum kiharae seeds with a unique 10-cysteine motif. FEBS J..

[21] Rogozhin E.A., Oshchepkova Y.I., Odintsova T.I., Khadeeva N.V., Veshkurova O.N., Egorov T.A., Grishin E.V., Salikhov S.I. (2011). Novel antifungal defensins from *Nigella sativa* L. seeds. Plant Physiol. Biochem..

[22] Vasilchenko A.S., Smirnov A.N., Zavriev S.K., Grishin E.V., Vasilchenko A.V., Rogozhin E.A. (2017). Novel Thionins from Black Seed (*Nigella sativa* L.) Demonstrate Antimicrobial Activity. Int. J. Pept. Res. Ther..

[23] Barashkova A.S., Sadykova V.S., Salo V.A., Zavriev S.K., Rogozhin E.A. (2021). Nigellothionins from Black Cumin (*Nigella sativa* L.) Seeds Demonstrate Strong Antifungal and Cytotoxic Activity. Antibiotics.

[24] Oshchepkova Y.I., Veshkurova O.N., Rogozhin E.A., Musolyamov A.K., Smirnov A.N., Odintsova T.I., Egorov T.A., Grishin E.V., Salikhov S.I. (2009). Isolation of the lipid-transporting protein Ns-LTP1 from seeds of the garden fennel flower (*Nigella sativa*). Russ. J. Bioorg. Chem..

[25] Barashkova A.S., Rogozhin E.A. (2020). Isolation of antimicrobial peptides from different plant sources: Does a general extraction method exist?. Plant Methods.

[26] Park C.J.C.B., Park C.J.C.B., Hong S.-S.S., Lee H.-S.S., Lee S.Y., Kim S.C. (2000). Characterization and cDNA cloning of two glycine- and histidine-rich antimicrobial peptides from the roots of shepherd’s purse, Capsella bursa-pastoris. Plant Mol. Biol..

[27] Beliaev D., Rogozhin E.A., Meleshin A.A., Tereshonok D.V., Derevyagina M.K., Yuorieva N.O., Tashlieva I.I., Djalilov F.S., Voronkova E. (2020). NsD3, a Defensin from *Nigella sativa*, Confers High Resistance of Several Commercial Potato Varieties to Fungi and Bacteria. Vitr. Cell Dev. Biol..

[28] Yeats T.H., Rose J.K.C. (2008). The biochemistry and biology of extracellular plant lipid-transfer proteins (LTPs). Protein Sci..

[29] Fleury C., Gracy J., Gautier M.F., Pons J.L., Dufayard J.F., Labesse G., Ruiz M., De Lamotte F. (2019). Comprehensive classification of the plant non-specific lipid transfer protein superfamily towards its sequence–structure–function analysis. PeerJ.

[30] Silverstein K.A.T., Graham M.A., Paape T.D., Vandenbosch K.A. (2005). Genome Organization of More Than 300 Defensin-Like Genes in Arabidopsis. Plant Physiol..

[31] Mergaert P., Nikovics K., Kelemen Z., Maunoury N., Vaubert D., Kondorosi A., Kondorosi E. (2003). A Novel Family in Medicago truncatula Consisting of More Than 300 Nodule-Specific Genes Coding for Small, Secreted Polypeptides with Conserved Cysteine Motifs. Plant Physiol..

[32] Meng C., Yan Y., Liu Z., Chen L., Zhang Y., Li X., Wu L., Zhang G., Wang X., Ma Z. (2018). Systematic analysis of cotton non-specific lipid transfer protein family revealed a special group that is involved in fiber elongation. Front. Plant Sci..

[33] Boutrot F., Guirao A., Alary R., Joudrier P., Gautier M.F. (2005). Wheat non-specific lipid transfer protein genes display a complex pattern of expression in developing seeds. Biochim. Biophys. Acta Gene Struct. Expr..

[34] De Samblanx G.W., Goderis I.J., Thevissen K., Raemaekers R., Fant F., Borremans F., Acland D.P., Osborn R.W., Patel S., Broekaert W.F. (1997). Mutational analysis of a plant defensin from radish (*Raphanus sativus* L.) reveals two adjacent sites important for antifungal activity. J. Biol. Chem..

[35] van der Weerden N.L., Anderson M.A. (2013). Plant defensins: Common fold, multiple functions. Fungal Biol. Rev..

[36] Slavokhotova A.A., Odintsova T.I., Rogozhin E.A., Musolyamov A.K., Andreev Y.A., Grishin E.V., Egorov T.A. (2011). Isolation, molecular cloning and antimicrobial activity of novel defensins from common chickweed (*Stellaria media* L.) seeds. Biochimie.

[37] Yang X., Li J., Li X., She R., Pei Y. (2006). Isolation and characterization of a novel thermostable non-specific lipid transfer protein-like antimicrobial protein from motherwort (*Leonurus japonicus* Houtt) seeds. Peptides.

[38] Terras F.R.G., Goderis I.J., Van Leuven F., Vanderleyden J., Cammue B.P.A., Broekaert W.F. (1992). In Vitro Antifungal Activity of a Radish (*Raphanus sativus* L.) Seed Protein Homologous to Nonspecific Lipid Transfer Proteins. Plant Physiol..

[39] Akkerdaas J., Finkina E.I., Balandin S.V., Santos Magadán S., Knulst A., Fernandez-Rivas M., Asero R., Van Ree R., Ovchinnikova T.V. (2011). Lentil (*Lens culinaris*) lipid transfer protein Len c 3: A novel legume allergen. Int. Arch. Allergy Immunol..

[40] Regente M.C., Giudici A.M., Villalaín J., De La Canal L. (2005). The cytotoxic properties of a plant lipid transfer protein involve membrane permeabilization of target cells. Lett. Appl. Microbiol..

[41] Souza A.A., Costa A.S., Campos D.C.O., Batista A.H.M., Sales G.W.P., Nogueira N.A.P., Alves K.M.M., Coelho-de-Souza A.N., Oliveira H.D. (2018). Lipid transfer protein isolated from noni seeds displays antibacterial activity in vitro and improves survival in lethal sepsis induced by CLP in mice. Biochimie.

[42] Molina A., Segura A., García-Olmedo F. (1993). Lipid transfer proteins (nsLTPs) from barley and maize leaves are potent inhibitors of bacterial and fungal plant pathogens. FEBS Lett..

[43] Bogdanov I.V., Shenkarev Z.O., Finkina E.I., Melnikova D.N., Rumynskiy E.I., Arseniev A.S., Ovchinnikova T.V. (2016). A novel lipid transfer protein from the pea Pisum sativum: Isolation, recombinant expression, solution structure, antifungal activity, lipid binding, and allergenic properties. BMC Plant Biol..

[44] Sarowar S., Kim Y.J., Kim K.D., Hwang B.K., Ok S.H., Shin J.S. (2009). Overexpression of lipid transfer protein (LTP) genes enhances resistance to plant pathogens and LTP functions in long-distance systemic signaling in tobacco. Plant Cell Rep..

[45] Molina A., Mena M., Carbonero P., García-Olmedo F. (1997). Differential expression of pathogen-responsive genes encoding two types of glycine-rich proteins in barley. Plant Mol. Biol..

[46] Maldonado A.M., Doerner P., Dixonk R.A., Lamb C.J., Cameron R.K. (2002). A putative lipid transfer protein involved in systemic resistance signalling in Arabidopsis. Nature.

[47] Wang C., Gao H., Chu Z., Ji C., Xu Y., Cao W., Zhou S., Song Y., Liu H., Zhu C. (2021). A nonspecific lipid transfer protein, StLTP10, mediates resistance to *Phytophthora infestans* in potato. Mol. Plant Pathol..

[48] Rogozhin E.A., Vasilchenko A.S., Barashkova A.S., Smirnov A.N., Zavriev S.K., Demushkin V.P. (2020). Peptide extracts from seven medicinal plants discovered to inhibit oomycete phytophthora infestans, a causative agent of potato late blight disease. Plants.

[49] Portz D., Koch E., Slusarenko A.J. (2008). Effects of garlic (*Allium sativum*) juice containing allicin on *Phytophthora infestans* and downy mildew of cucumber caused by Pseudoperonospora cubensis. Eur. J. Plant Pathol..

[50] Rogozhin E.A., Ryazantsev D.Y., Grishin E.V., Egorov T.A., Zavriev S.K. (2012). Defense peptides from barnyard grass (*Echinochloa crusgalli* L.) seeds. Peptides.

[51] Wang X., Bunkers G.J., Walters M.R., Thoma R.S. (2001). Purification and Characterization of Three Antifungal Proteins from Cheeseweed (*Malva parviflora*). Biochem. Biophys. Res. Commun..

[52] Galindo-Luján R., Pont L., Minic Z., Berezovski M.V., Sanz-Nebot V., Benavente F. (2021). Characterization and differentiation of quinoa seed proteomes by label-free mass spectrometry-based shotgun proteomics. Food Chem..

[53] Cox J., Neuhauser N., Michalski A., Scheltema R.A., Olsen J.V., Mann M. (2011). Andromeda: A peptide search engine integrated into the MaxQuant environment. J. Proteome Res..

[54] Rogozhin E.A., Slezina M.P., Slavokhotova A.A., Istomina E.A., Korostyleva T.V., Smirnov A.N., Grishin E.V., Egorov T.A., Odintsova T.I. (2015). A novel antifungal peptide from leaves of the weed *Stellaria media* L. Biochimie.

