# Supplementary material for: Diversity of Cationic Antimicrobial Peptides in Black Cumin (Nigella sativa L.) Seeds
Source: Int J Mol Sci. 2023 Apr 29;24(9):8066. doi: 10.3390/ijms24098066 (PMC10179141; doi:10.3390/ijms24098066)
Supplement: Supplementary file 1 [file ijms-24-08066-s001.zip › Supplemental Figure S1.pdf]

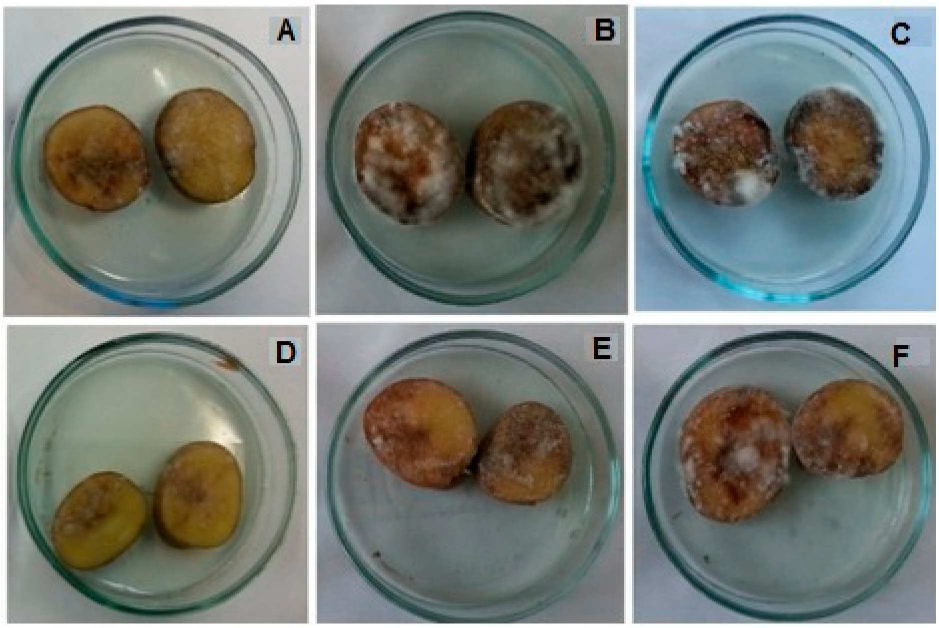

Figure S1. Development of *P. infestans* isolates on potato tuber discs (susceptible isolate OSV12, A, B, C) and (resistant isolate PRIL, D, E, F) at the maximum concentration of NsLTP2: A, D - after 4 days of incubation; B, E - after 5 days of incubation; C, F - after 6 days of incubation.
