# Supplementary material for: Diversity of Cationic Antimicrobial Peptides in Black Cumin (Nigella sativa L.) Seeds
Source: Int J Mol Sci. 2023 Apr 29;24(9):8066. doi: 10.3390/ijms24098066 (PMC10179141; doi:10.3390/ijms24098066)
Supplement: Supplementary file 1 [file ijms-24-08066-s001.zip › Supplementary file S2.pdf]

Supplementary

Antifungal activity of NsLTP3			
	A. niger VKM F-33	*B. sorokiniana* VKM F-1448	*B. cinerea* TSKHA isolate
NsLTP3 IC_{50}, µM	1.05	1.55, plasmolysis	1.88

Antibacterial activity of *N. sativa* LTPs					
	Pseudomonas syringae (G–)	*Xanthomonas campestris* (G–)	*Pectobacterium carotovorum* (G–)	*Bacillus subtilis* (G+)	*Clavibacter michiganense sb sp. michiganense* (G+)
NsLTP1	10.0±1.0	No effect	No effect	7.0±0.5	No effect
NsLTP3	11.5±1.3	7.0±0.5	12.0±1.4	8.4±1.0	11.2±1.1
