# Supplementary figures and images for: Diversity of Cationic Antimicrobial Peptides in Black Cumin (Nigella sativa L.) Seeds
Source: Int J Mol Sci. 2023 Apr 29;24(9):8066. doi: 10.3390/ijms24098066 (PMC10179141; doi:10.3390/ijms24098066)

Current Chromatogram(s)

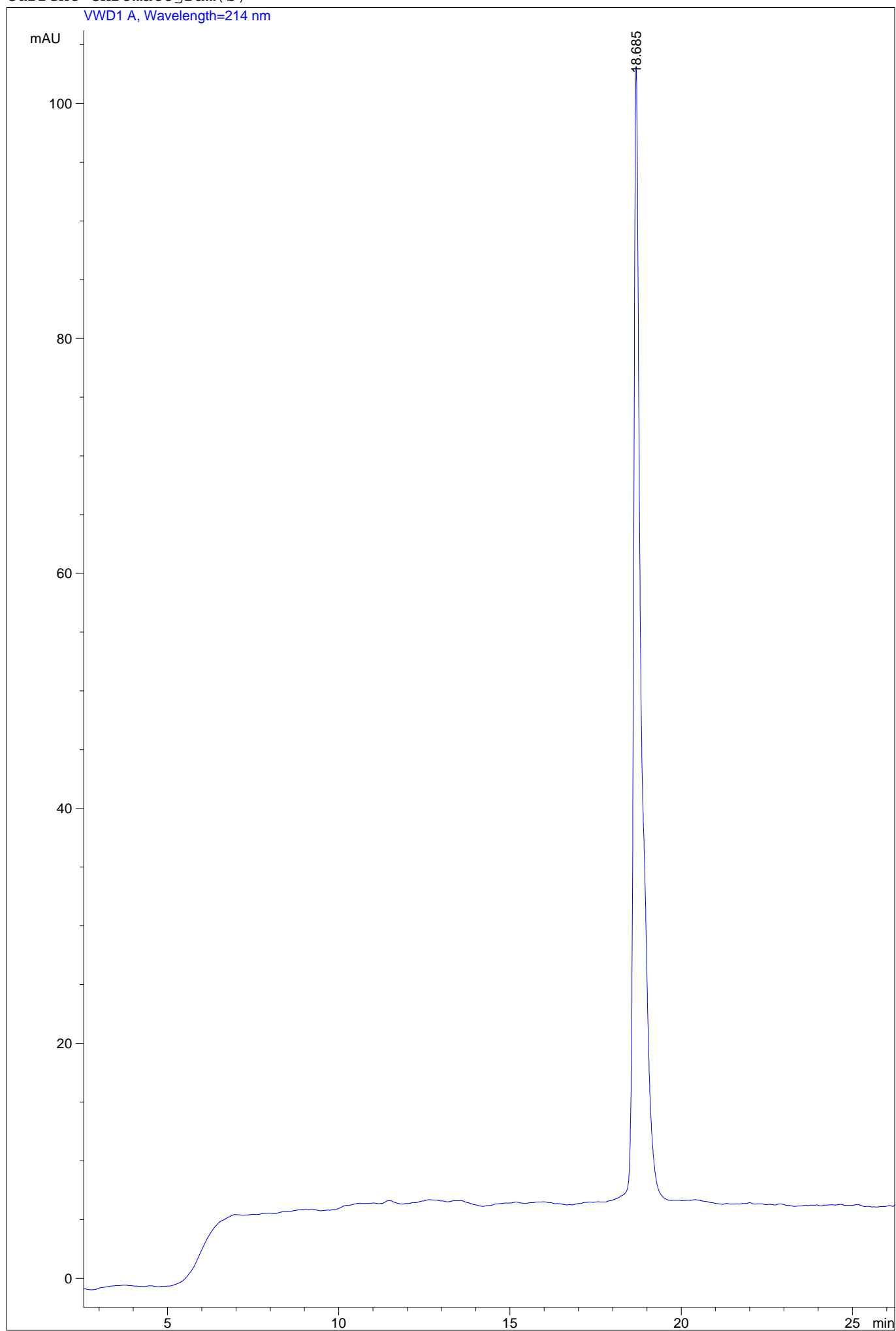

Supplement: Supplementary file 1 [file ijms-24-08066-s001.zip › NsD4.pdf]

Current Chromatogram(s)

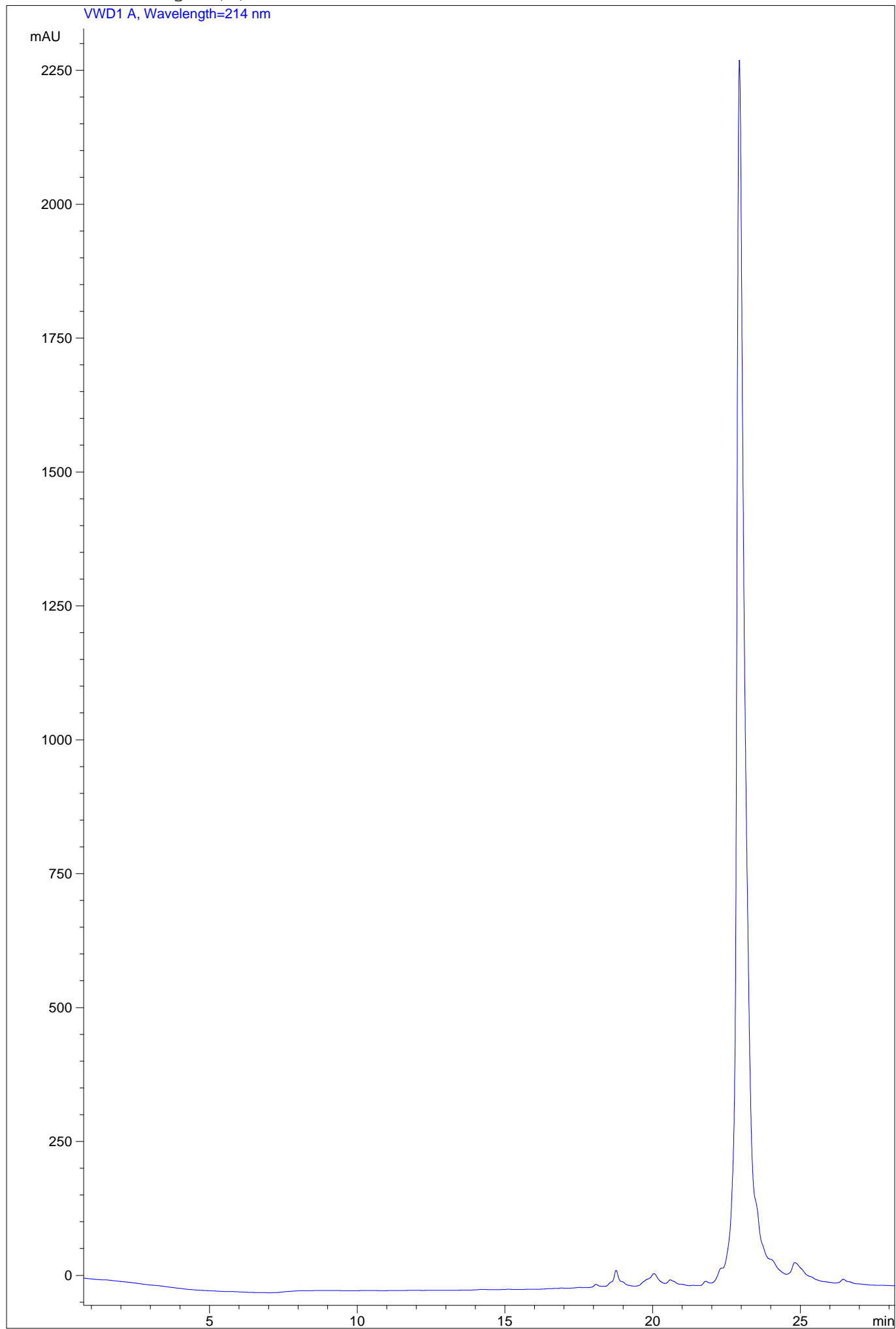

Supplement: Supplementary file 1 [file ijms-24-08066-s001.zip › NsLTP2.pdf]

Current Chromatogram(s)

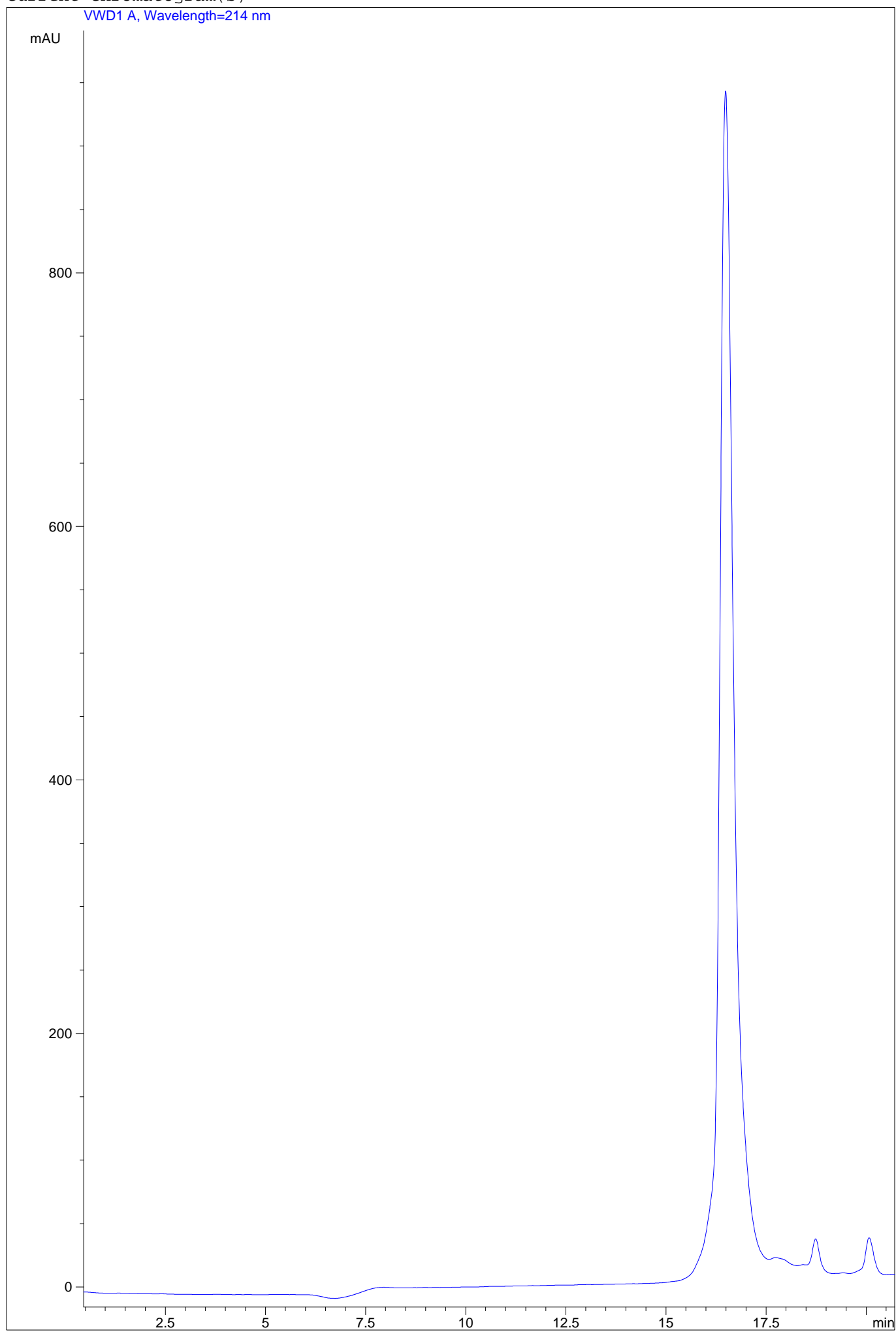

Supplement: Supplementary file 1 [file ijms-24-08066-s001.zip › NsLTP3.pdf]
